# Endurance sport and cardiovascular health

**DOI:** 10.1186/2052-1847-7-S1-O13

**Published:** 2015-08-11

**Authors:** Sanjay Sharma

**Affiliations:** 1 Department of Cardiovascular Sciences, St George’s University of London, Cranmer Terrace, London, SW17 0RE, UK

## 

The cardiovascular benefits of exercise are established and individuals exercising regularly reduce their risk of adverse events from coronary artery disease by 50% and gain at least 3 additional years of life. When one considers the burgeoning epidemic of childhood obesity and its complications, exercise may be regarded as the most clinically and cost effective prescription dispensed by healthcare professionals. The intensity of exercise required to achieve these benefits is 6-8 METS which equates to walking briskly or jogging for 30 minutes daily. Competitive athlete, particularly those engaging in endurance events such as long distance running, cycling, rowing, canoeing, triathlon, marathon and iron man events partake in physical activity of a much higher intensity for as many as 20 hours per week.

The requirement of a 5-6 fold increase in cardiac output is met by physiological structural and functional adaptations within the heart. Sinus bradycardia, large QRS complexes and a 15-20% increase in ventricular cavity size are the hall mark of the ‘athlete’s heart’ and are most pronounced in large male endurance athletes. Up to 50% of male endurance athletes show ventricular dimensions exceeding predicted upper limits for the general population. Such alterations are considered benign and reverse after a period of detraining.

The potential hazards of intensive exercise are occasionally highlighted by the sudden cardiac death of an individual during or immediately after exercise, however, such catastrophes are extremely rare, largely striking young athletes harboring inherited or congenital arrhythmogenic substrates. Despite the emotive visibility afforded by these deaths in the media, the positive reputation of exercise remains unscathed by the fact that exercise is regarded as a mere trigger for arrhythmia in a small proportion of predisposed individuals but never directly incriminated in the pathogenesis of the fatal substrate.

The past 2 decades has witnessed an extraordinarily engaging athletic milieu where humanly possible achievements seem infinite. In parallel there have been multiple reports of increased serum concentrations of biomarkers of cardiac damage and transient cardiac dysfunction after intensive endurance exercise. The mechanisms for these observations is uncertain but raise the possibility that repeated bouts of intensive endurance exercise may culminate in adverse cardiac remodeling and arrhythmogenic substrates in a small minority of endurance athletes. Perhaps the most persuasive evidence for this concern is the 5-fold increase in the prevalence of atrial fibrillation in middle aged endurance athletes compared with the relatively sedentary population of the same age.

